# Promising development from translational or perhaps anti-translational research in breast cancer

**DOI:** 10.1186/2001-1326-1-17

**Published:** 2012-08-28

**Authors:** Michael Retsky, Romano Demicheli, William JM Hrushesky, Patrice Forget, Marc De Kock, Isaac Gukas, Rick A Rogers, Michael Baum, Katharine Pachmann, Jayant S Vaidya

**Affiliations:** 1 Harvard School of Public Health, BLDG I, Rm 1311, 665 Huntington, Ave, Boston, MA, 02115, USA; 2 Royal Free and UCL Medical School, Centre for Clinical Science and Technology, University College London, Clerkenwell Building, Archway Campus, Highgate Hill, London, UK; 3 Scientific Directorate, Fondazione IRCCS Istituto Nazionale Tumori, Via Venezian 1, 20133, Milan, Italy; 4 Oncology Analytics, Inc, 8751 W. Broward Blvd, Suite 500, Plantation, FL, 33324, USA; 5 Department of Anesthesiology, Universite Catholique de Louvain, St-Luc Hospital, av. Hippocrate 10-1821, 1200, Brussels, Belgium; 6 James Paget University Hospital, Lowestoft Road, Gorleston, Great Yarmouth, Norfolk, NR31 6LA, UK; 7 Royal Free and UCL Medical School, Centre for Clinical Science and Technology, University College London, Clerkenwell Building, Archway Campus, Highgate Hill, London, N19 5LW, UK; 8 Department of Experimental Hematology and Oncology, Clinic for Internal Medicine II, Friedrich Schiller University, 07747, Jena, Germany; 9 Clinical Trials Group of the Division of Surgery and Interventional Science, University College London, Clerkenwell Building, Archway Campus, Highgate Hill, London, N19 5LW, UK

**Keywords:** Breast cancer, Early relapses, NSAID, Perioperative ketorolac, Inflammatory oncotaxis, Angiogenesis, Dormancy, Transient systemic inflammation

## Abstract

**Background:**

A great deal of the public’s money has been spent on cancer research but demonstrable benefits to patients have not been proportionate. We are a group of scientists and physicians who several decades ago were confronted with bimodal relapse patterns among early stage breast cancer patients who were treated by mastectomy. Since the bimodal pattern was not explainable with the then well-accepted continuous growth model, we proposed that metastatic disease was mostly inactive before surgery but was driven into growth somehow by surgery. Most relapses in breast cancer would fall into the surgery-induced growth category thus it was highly important to understand the ramifications of this process and how it may be curtailed. With this hypothesis, we have been able to explain a wide variety of clinical observations including why mammography is less effective for women age 40–49 than it is for women age 50–59, why adjuvant chemotherapy is most effective for premenopausal women with positive lymph nodes, and why there is a racial disparity in outcome.

**Methods:**

We have been diligently looking for new clinical or laboratory information that could provide a connection or correlation between the bimodal relapse pattern and some clinical factor or interventional action and perhaps lead us towards methods to prevent surgery-initiated tumor activity.

**Results:**

A recent development occurred when a retrospective study appeared in an anesthesiology journal that suggested the perioperative NSAID analgesic ketorolac seems to reduce early relapses following mastectomy. Collaborating with these anesthesiologists to understand this effect, we independently re-examined and updated their data and, in search of a mechanism, focused in on the transient systemic inflammation that follows surgery to remove a primary tumor. We have arrived at several possible explanations ranging from mechanical to biological that suggest the relapses avoided in the early years do not show up later.

**Conclusions:**

We present the possibility that a nontoxic and low cost intervention could prevent early relapses. It may be that preventing systemic inflammation post surgery will prevent early relapses. This could be controlled by the surgical anesthesiologist’s choice of analgesic drugs. This development needs to be confirmed in a randomized controlled clinical trial and we have identified triple negative breast cancer as the ideal subset with which to test this. If successful, this would be relatively easy to implement in developing as well as developed countries and would be an important translational result.

## Background

We are an eclectic group of scientists and physicians who have been working for almost two decades on an unusual research project dealing with breast cancer. In this project we are not conducting laboratory research nor are we treating patients with experimental therapies. We are deconstructing clinical data using powerful numerical and computational tools and trying to understand what was the actual tumor growth activity that could have produced these data. We are then extrapolating these findings to predict new methods of therapy that take advantage of the extraordinary temporal patterns of tumor growth and spread. It has been brought to our attention that this is leading toward translational research which according to Wang and Marincola represents the need to find cost-effective solutions to the treatment of chronic diseases which represents 2/3 of health care spending in most countries [1]. Translational research is a way of thinking about and conducting scientific research to make the results of research applicable to the population under study. In the field of medicine, for example, it is used to translate the findings in basic research more quickly and efficiently into medical practice and, thus, meaningful health outcomes, whether those are physical, mental, or social. Translational is a label for a research approach that seeks to move “from bench to bedside” or from laboratory experiments through clinical trials to actual point-of-care patient applications (from Wikipedia). We are translators starting from clinical findings, looking at biology and then coming back to clinics. Our manner of investigation is significantly closer to the actual disease course than research that starts from basic research, at least in our opinion.

It is ironic that, as described later, we have also been accused of publishing research that was anti-translational in breast cancer.

**Figure 1:**
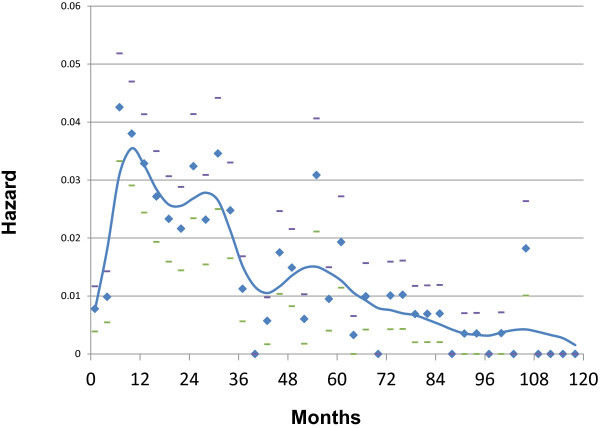
** Hazard of relapse for premenopausal patients treated at *Istituto Nazionale Tumori* in Milan, Italy.** Hazard is the number of events that occur in a time interval divided by the number of patients who enter that time as event free. Patients were treated by mastectomy well before the routine use of adjuvant therapy. The time interval in all hazard figures used here is 3 months. Average and standard deviations are indicated as diamonds and bars. The curve was obtained by a kernel-like smoothing procedure.

**Figure 2:**
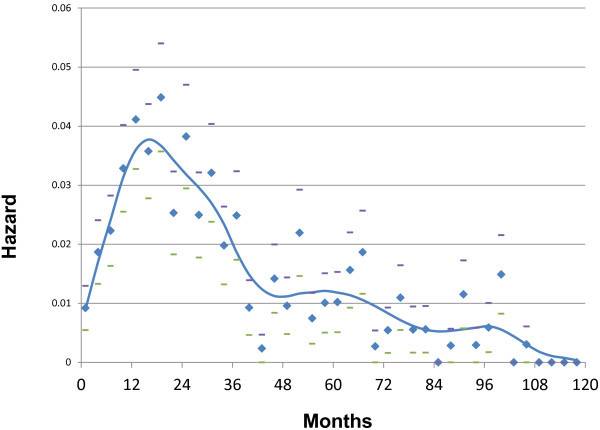
** Hazard of relapse for postmenopausal patients treated at *Istituto Nazionale Tumori* in Milan, Italy.** Definitions are the same as indicated in Figure 1 but the patient population is postmenopausal.

Our project started off in the 1990s when two reports were published from different countries describing bimodal relapse patterns among women treated for early stage breast cancer [2,3]. That is, after breast cancer is discovered and surgically removed, the real danger is that it will come back in other organs such as lung, liver, bones and brain where treatment is much less successful. That is the common path leading to breast cancer death. In these new data, there was an early wave of relapses in the first four years and then a period when very few such events happened. This was then followed by a second much broader wave of relapses that peaked at about 6 years and extended to 10–15 or more years. Milan data that we have analyzed in detail are shown in Figures 1 and 2. A difference between pre- and post-menopausal relapse hazard may be seen. Bimodal patterns have now been identified in 20 independent breast cancer databases from US, Europe and Asia [4]. This is apparently not restricted to breast cancer as we have noted similar recurrence dynamics among patients who are resected for primary control of prostate, lung, and pancreatic cancers, as well as osteosarcoma and melanoma [5-11]. 

The most extraordinary part is that the early wave occurs at a constant period after surgery, irrespective of the stage of the disease, which cannot be explained by the conventional ideas of spread of solid tumors. The magnitude, but not the timing of this wave is related to clinical factors. These recurrence dynamics were not explainable using the then well-accepted continuous growth description for primary and metastatic tumors nor did it agree with the analysis of local relapse occurrences [12]. Conventional models would suggest that relapse should occur earlier for advanced stages and vice versa.

We thereby reasoned that something was clearly wrong with the continuous growth description. This was not just an academic issue. Tumor growth was commonly considered to be described by the damped exponential Gompertz equation [13-16]. That is, rapid exponential growth when it is small but gradually slowing as the tumor enlarges. Since chemotherapy is most effective when tumors are growing rapidly, the way to use these drugs is to assume metastatic cancer is present just after surgery but too small to be detected yet. If it is there at all, it is growing at the most rapid rate possible and thus most responsive to cytotoxic drugs that interfere with cellular proliferation and/or division. The strategy then is to treat intensely as soon as possible up to the limits of tolerability and then stop and hope that any microscopic tumors were eradicated. The conventional adjuvant treatment model is not much more complicated than that [16]. It works some of the time but not nearly often enough to satisfy either patients or their physicians.

Our project was initially supported by a small grant to make a computer simulation of the Milan data to create an expert system with which we could develop prognostic tools and hopefully design new therapies. Based on computer simulation, we eventually proposed that tumor growth was far from continuous but rather intermittent and dynamically non-random in some important ways. We found that most relapses in breast cancer are the result of sudden metastatic growth that starts at or about the time of primary tumor excision. That is, something happens around the time of surgery that kicks off growth of tumors resulting in relapses within four years.

This is all described in our several reviews that are available freely on-line and does not need to be extensively discussed here [4,17,18]. To summarize, we proposed that metastatic tumor growth is mostly inactive before primary surgery but much happens afterwards. The surgery-synchronized early relapses include new growth from single cells and surgery-induced angiogenesis from existing dormant avascular micrometastases. The late relapses (> 4 years) are not synchronized to surgery and occur gradually for the remainder of the lives of patients at risk.

Between 50% and 80% of relapses result from surgery initiated growth. This predominant proportion increases with the size of the primary tumor. For tumors < 1 cm in diameter, 50% of those tumors destined to recur, did so very early. For larger tumors the effect increased to 80%. Surgery-induced angiogenesis of dormant avascular micrometastases occurred in 20% of premenopausal women with positive lymph nodes. This mode of relapse also occurred among other categories of patients. It was 2:1 more common for premenopausal compared to postmenopausal and occurred five times more frequently among node positive contrasted to node negative ones. In premenopausal patients, timing of surgery within the menstrual cycle impacts outcome according to some investigations [19]. While this concept still needs confirmation from well-designed randomized clinical trials, it points out the importance of the host/tumor environment at the time of surgery [20,21].

There are indications that this general effect was recognized long ago. Remarkably, surgeons 2000 years ago were able to remove breast tumors and many patients survived the surgery. Aulus Cornelius Celsus (30BC – 38 AD) wrote: “First there is the cacoethes, then carcinoma without ulceration, then the fungating ulcer. None of these can be removed but the cacoethes: the rest are irritated by every method of cure. The more violent the operations the more angry they grow. Some use caustics, some burning iron, others remove the growth with the scalpel. After excision, even though a cicatrix is formed, it recurs, bringing with it the cause of death, whereas at the same time, most people, by using no violent methods to attempt the extirpation of the disease but only applying mild medications to soothe it, protract their lives, notwithstanding the disorder, to an extreme old age.” This is of course relative to the life expectancy in those times. Galen (131 – 203 AD) wrote: “We have often cured this disease in the early stages, but after it has grown to a noticeable size no one has cured it with surgery” [22]. So we are certainly not the first to notice that removing breast tumors can sometimes accelerate disease and that this increases with tumor size.

The most important findings of our early work are that something happens at or about the time of surgery to accelerate or induce metastatic activity that results in early relapses. These early relapses comprise over half of all relapses. Surgery-induced angiogenesis of dormant avascular micrometastases and surgery-induced activity of single malignant cells are implicated. Late relapses are apparently not synchronized to the time of surgery.

How does this experience fit in terms of translational cancer research? Children’s Hospital and Harvard Medical School considered it a significant scientific development and with Judah Folkman’s approval issued a press release when one of our papers was published in International Journal of Surgery [23].

In that paper, we reported that we could explain the controversial results of early detection of breast cancer for women age 40–49. The probability of surviving breast cancer decreases dramatically as the primary tumor size and the number of axillary lymph nodes positive for cancer increases. Since a patient diagnosed with large tumor and multiple lymph nodes logically could have been diagnosed with smaller tumor and fewer positive nodes at some time in the past, it made perfect sense that detection at an earlier time would have resulted in improved outcome. The amount of benefit was not known so, beginning in the 1960s, clinical trials of early detection were initiated in US, UK and Sweden. As data were emerging years later, it was clear that for women age 50–59 there was a strong and early appearing 15 to 20% mortality advantage to screening mammography.

When data for women age 40–49 were examined, however, there was a clear and wholly unexpected mortality excess in the intervention arms. This excess mortality lasted for six to eight years in the various randomized controlled trials, but was clearly present. After this six to eight year span, some benefit showed up. This caused high anxiety and scientific and emotional consternation. At a NIH consensus conference called on the subject, an expert panel concluded that early detection should begin at age 50 years. After much argument, a second expert panel was then formed that concluded mammography should begin at age 40. Arguments ensued that are still continuing years later.

We stepped into this ruckus by publishing that the Milan data suggest surgery-induced angiogenesis for premenopausal node positive patients would produce 1 early relapse per 10,000 persons age 40–49 who were screened for early detection. The deaths from this effect would appear 2–3 years after the start of the trial. This was very close to what was reported in trials and overviews. Our findings agreed with trial data that early detection worked better for postmenopausal women than it did for premenopausal women and may in fact harm some young women.

This got much attention including a 1200 word write-up in the Wall Street Journal by Pulitzer Prize winning reporter Amy Marcus [24]. Our findings at this juncture were good science but not what some people wanted to hear. Our findings were, in some peoples’ opinions, a step backward in their efforts to get women screened with mammography starting at age 40 (or even younger). In their opinions, this was opposite to translational research. It showed early detection - the highly accepted way to improve outcome in breast cancer – could in some cases paradoxically result in earlier death than if they were not screened. There were many angry people and they were angry at us and any others who openly discussed the paradox [25]. Mammography and the resulting biopsies, histopathologic, biochemical and genetic analyses of tissue samples and extensive additional imaging resulting represent very big and profitable business for medical practitioners and various connected medical-industrial institutions. Of course other advocates of mammography starting at age 40 were motivated by the most altruistic of reasons fully believing early detection saves lives and wanting this to be universally practiced.

We attempted to steer clear of producing clinical uncertainty [26]. "We say this is indirect evidence; we think this is a key to understanding the biology of breast cancer," Retsky told WebMD. "We certainly do not suggest any changes in clinical practice based on this. We hope this will entice clinical and experimental people to test these hypotheses."

Without going into too many details, we nonetheless got dragged into the mammography wars and it became quite an unpleasant situation. “Don’t believe any of this” came as a quoted comment from the director of screening for American Cancer Society [26]. In addition, there were emails from a prominent mammography proponent asking Dr. Folkman, who was coauthor of one of our papers, to remove his name as coauthor so as to reduce credibility of our papers suggesting surgery can sometimes accelerate metastatic tumor growth. There was an angry and insulting letter to the editor of a journal that published one of our papers asking why the paper in question did not get picked up by peer reviewers as obviously wrong [27]. The Director of the National Cancer Institute declared that the mammography controversy is over [28]. The US Senate voted 98–0 that mammography should be routine for women age 40–49 [29].

Meanwhile, we were publishing papers and submitting grant proposals to support our research. As evidence of the soundness of our findings, we naively reported that based on clinical data we could quantitatively explain the mammography paradox. But this tactic apparently backfired. Our proposals did not survive triage. As an apparent result, there was an unpleasant span of years during which we had no financial support. This was a low spot in our research. The simulation research that was conducted in the US was halted (but the clinical research continued in Italy) and we continued to publish papers. We report this since researchers intending to conduct translational research should be aware that their findings might intentionally or unintentionally trespass on sensitive terrain with unfavorable results.

There was one very important favorable outcome from the publicity on our papers that needs to be mentioned. Two of the letters sent to Editor of International Journal of Surgery in response to our paper led us into a new research topic. One was from an African American attorney who asked if what we reported could explain why African Americans commonly say that “cancer spreads when the air hits it” or words to that effect [30]. The other letter was from Dr. Isaac Gukas who is a breast surgeon in UK but spent 15 years practicing oncology in his native Nigeria [31]. Dr. Gukas noted that in Nigeria it was typical for women to present in their early 40s with breast cancer but often decline treatment by a physician, instead seeing a traditional practitioner who would prescribe herbal remedies. Their fear was that surgery would “provoke the cancer”. When eventually these persons went to a physician it was often with locally advanced disease. Outcome after intervention was poor.

It did not take us long to discover that African Americans present with breast cancer at average age 46 years while European Americans present at average age 57 years. Thus African American breast cancer is mostly premenopausal while European American breast cancer is mostly postmenopausal. This train of thought led us to write a number of papers with Dr. Gukas as co-author proposing an explanation of the racial disparity in breast cancer outcome based on this now expanding theory [32-34]. It is quite possible that if most trials of mammography were conducted in Nigeria instead of Sweden, there would have been little if any benefit shown and mammography would not be a common practice today.

Getting back to breast cancer research, we are now funded again and vigilantly searching for new data that will teach us more about surgery-induced tumor activity and that may ultimately lead to improved post-resection breast cancer outcomes.

## Results

There has been an important recent development. A paper was published in an anesthesiology journal in June 2010 that reported on a retrospective study of 327 consecutive mastectomy patients treated in one Brussels hospital and by one surgeon [35]. Normally, anesthesiologists have a 30-day window of interest. If the patients are alive and as well as can be expected 30 days after a surgical intervention, the anesthesiologist considers his or her job done satisfactorily. In the Brussels report however the patients were examined for metastatic relapse events for up to 4 years post surgery and grouped by what analgesic drug was used perioperatively.

Approval of the Ethical Committee of St-Luc Hospital was provided by the Commission d'Ethique Biomédicale Hospitalo-Facultaire de l'Université catholique de Louvain (CEBH) of the Université Catholique de Louvain (Brussels, Belgium), Chairperson Prof Dr. J.M. Maloteaux. Investigators were unable to obtain consent from the patients for this retrospective study and the need for written informed consent from participants was waived, as accepted by the CEBH.

Patients were treated with mastectomy and conventional adjuvant therapy. Chemotherapy, radiotherapy, and endocrine therapy were performed according to international expert consensus protocols (9th and 10th St-Gallen consensus). Follow-up in that initial report was average 27.3 months with range 13–44 months. Patients who received anti-inflammatory drugs were compared with those who had not and their hazard of recurrence was analyzed and compared. We now report an independent update of those data as of September 2011.

**Figure 3:**
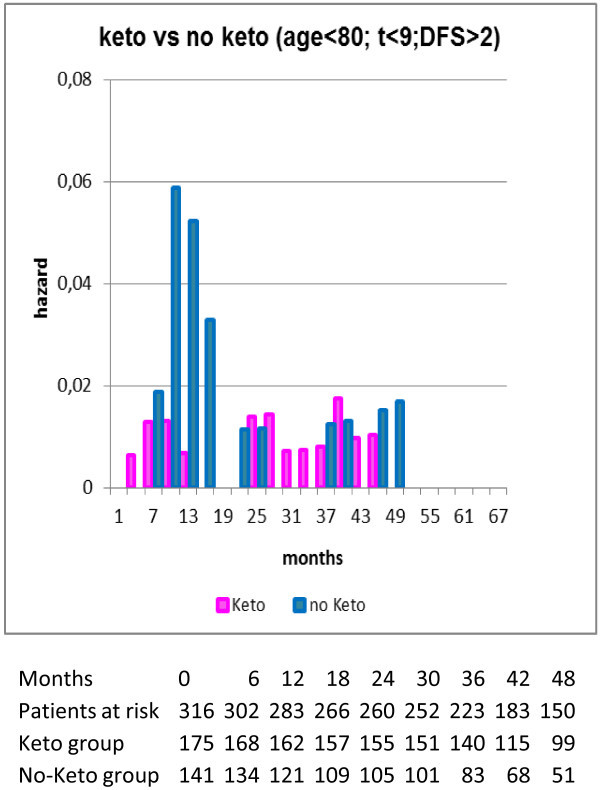
** Forget et al. data were updated September 2011 and shown in hazard form.** Patient data are presented in the table. Patients included in this figure were less than 80 years of age, tumor less than 9 cm diameter and disease free survival greater than 2 months. It can be seen that relapses in months 9–18 accounted for the major difference between ketorolac and non-ketorolac patients. Data were provided by Sarah Amar and analyzed by Romano Demicheli.

The startling result was that one low cost drug- the non steroidal anti-inflammatory (NSAID) ketorolac – was strongly associated with far better four year breast cancer outcome than any of the other three non-NSAID analgesics (sufentanil, clonidine, and ketamine). The early relapses that we have associated with surgery are all but absent among patients who had been given perioperative ketorolac [36]. These data from the Brussels study are shown as updated September 2011 in Figure 3. 

## Discussion

Even with the insight of simulations, it is sometimes impossible to determine with certainty what happened to each of the various relapse modes in a particular report. However in this case, it appears that perisurgical ketorolac may dramatically reduce the initiation of surgery-induced angiogenesis as well as single cell proliferation after surgery. If this observation holds up to further scrutiny, it could mean that the simple use of this safe and effective anti-inflammatory agent at the time of surgery might eliminate most early relapses.

Published along with the original Forget et al. study, an outline of a number of possible effects of surgery and anesthesia on cancer growth was presented by Gottschalk et al. [37]. These include stress, immunosuppression, pain, transfusion, inflammation, hypothermia, and a few others. In view of the extensive literature discussing connections and correlations between cancer growth and inflammation, our interest was drawn toward inflammation as possibly a key metastasis producing process.

Balkwill et al. write that if genetic damage is the “match that lights the fire” of cancer, then inflammation is the “fuel that feeds the flames” and that inflammation affects both the survival and proliferation of already initiated cancer cells [38]. Since Virchow first proposed in 1863 that tumors could originate from sites of chronic inflammation, it has been well established that chronic inflammation both contributes to cancer progression and predisposes tissue to various types of primary and metastatic cancer [39].

Based on Pascual et al. data from a colon cancer study, transient inflammation can also be both local and systemic [40]. They measured the proinflammatory cytokine interleukin-6 (IL-6) in serum prior to surgery and in peritoneal fluid during surgery to establish baseline IL-6, and again at 4, 12, 24 and 48 hours and at 4 days after surgery to determine a temporal trend. They found levels of IL-6 in serum at approximately 1/300 of the concentrations seen in peritoneal fluid. Judging by their data it would seem that levels in serum would gradually return to baseline in a week or so. While not breast cancer surgery we can assume that systemically and transiently something similar occurs in surgery to remove breast cancer.

The inflammatory response is initiated by tissue damage and can be intensified by mast cells, which release histamine, which then markedly increases the permeability of adjacent capillaries. The severity, timing, and local character of any particular inflammatory response depend on the cause, location and site of the area affected, and host’s condition [41]. Inflammatory oncotaxis, a term used to describe tumor growth at a site of inflammation, is occasionally seen in persons with known or occult cancer and who have local trauma [42-45]. (Note in particular the El Saghir et al. case report and the published comments [44].) Martins-Green et al. studied an avian system in which a virus is the carcinogenic agent [46]. When newly hatched chicks are given injections of Rous sarcoma virus, a tumor develops only at the site of injection unless a wound is made a distance away from the primary tumor where a tumor develops at the site of wounding. They found that when inflammation was inhibited, tumors were also inhibited; when inflammation could not be stopped, tumors developed as before.

**Figure 4:**
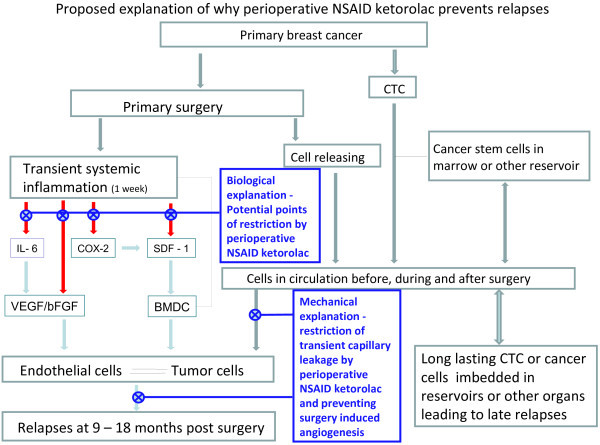
** Symbolic description of proposed mechanical and biological explanations for Forget et al. data.** Early relapses are assumed to be related, at least in part, to the inflammatory process due to primary tumor surgical removal, directly or indirectly eliciting peritumoral endothelial cell proliferation according to the biological mechanisms. A) Angiogenic factors, like VEGF and bFGF, are directly released or even produced via IL-6; B) Bone marrow derived CXCR-4 positive cells, acting both on tumor foci and on the inflammatory process, are mobilized by SDF-1, directly released or even produced via COX-2. Perioperative Ketorolac would restrict both endocrine and cellular pathways, thus impairing the metastatic process. CTC refers to circulating tumor cells. Also shown is how a mechanical explanation prevents these early relapses. Capillary leakage from transient systemic inflammation as a result of the surgery in the presence of circulating cancer cells and cells released during surgery and resulting inflammatory oncotaxis is blocked by directly preventing the inflammation. This prevents single cell activation. In addition, NSAIDS have antiangiogenic properties thus surgery-induced angiogenesis is prevented.

We have two categories of hypotheses that may explain Forget et al. data of outcome for patients treated with ketorolac or not. While there is some overlap, the first is more or less mechanical and the second is biological in nature. Figure 4 shows a schematic description of what we suspect to be at least some of the mechanisms governing metastatic relapse from early breast cancer.

### Mechanical explanation for Forget et al. data

It is well established that many cancer patients have circulating cancer cells [47,48]. Camara et al. have been studying circulating epithelial cells that might or might not be tumor cells. In 11 patients who were monitored before surgery and 10 minutes and 30 minutes after surgery, the immediate release of cells during surgery was analyzed. There was almost no change in cell numbers during this early time after surgery. However, in another group of 25 patients, cell numbers increased steeply by an average of over ten-fold in 85%of patients by the third day after surgery. There was a wide range from a slight decrease to 1000 fold increase. The average returned to nearly pre-surgery levels at the next measurement 7 days after surgery [49]. Such a delayed increase in what may be circulating cancer cells after breast cancer surgery was indicated in data by Daskalakis et al. [50].

Perhaps the transient systemic inflammation accompanying surgery and subsequent inflammatory oncotaxis is part of the metastatic tumor seeding process. It may be that what we previously called dormant single cells induced into metastatic growth were at least in some cases residing not at the site of eventual relapse. Rather, circulating cancer cells in an inflammatory environment extravasate, resulting a number of months later in a metastatic tumor. Circulating cancer cells are well documented. Surgical induction of inflammation is apparently universal. Capillary leakage is obviously enhanced by inflammation. While it depends on specific organs and physiologic conditions, blood flow in capillaries has been measured at approximately 0.5 mm/sec which could make leaky capillary venules a very efficient way for circulating cancer cells to enter tissue, thus reducing their concentration in circulation [51]. It is thereby logical to expect that an effective peri-surgical anti-inflammatory strategy may affect surgery-induced single cell activation and resulting cancer spread. There are only a few relapses in the ketorolac data shown in Figure 3 that appear to be surgery-induced angiogenesis events. This may be attributed to the reduced usage of opioids for pain management with ketorolac and also the antiangiogenic properties of NSAIDs [52,53].

### Biological explanation for Forget et al. data

The hypothesis that decreasing the inflammatory response to the surgical maneuver could interfere with the angiogenesis switch of avascular micrometastases is not outlandish. A few hypotheses can be put forward, including, but not limited to, the following. At steady-state conditions in adult mammals, most endothelial cells are quiescent and are believed to contribute to organ homeostasis and tumor dormancy [54]. However, in response to inflammation the upregulation and release of factors stimulating endothelial cells to proliferate could also induce endothelial cells to secrete specific cytokines that reciprocally support the regeneration of normal and malignant stem cells.

The metastatic process is believed to be supported by tumor stem cells, which are able to reproduce the cancer progeny. Tumor stem cells, as normal stem cells, require a supporting “niche”, i.e. a subset of tissue cells and extracellular substrates defining a specialized microenvironment that is able to modulate the stem cell function (quiescence or proliferation). The occurrence of a metastatic “vascular niche” where endothelial cells play a main role and where an angiogenesis dependent dormancy could result from the cross-talk between tumor cells and endothelial cells (perhaps by regulation of the Notch signaling) has been suggested [55]. If cancer stem cells need to interact with a vascular niche to express their potential, it is reasonable that the latter, under an angiogenic spike by the surgical approach to primary tumor, may appreciably contribute to dormancy interruption. If so, reducing inflammation could result in impairment of the dormant foci wake up process [56].

Tissue lesions induce mobilization of bone marrow derived cells that are capable of responding to chemoattractant signals from various organs, where they undergo a homing process and where they release several chemokines [57]. This phenomenon is prominent during neovascularization of wounded tissues via direct or paracrine activity inducing capillary formation. A common basis of the above-mentioned processes is cell trafficking. Indeed, while the intravascular dissemination of normal stem cells is essentially passive, mobilization from their usual niche and homing in a given tissue is regulated by specific signals. Hematopoietic stem cells, for example, express the chemokine receptor CXCR4 and selectively respond to SDF-1α. The SDF-1/CXCR4 axis is a main regulator of the normal cell trafficking underlying the tissue homeostasis. It is also involved in tumor cell trafficking as CXCR4 overexpression is known in more than 20 human tumor types, including ovarian, prostate, esophageal, melanoma, neuroblastoma, and renal cell carcinoma [58].

NSAIDs may interfere with SDF1 levels via the pathway COX-2 - > PGE - > SDF-1, thus resulting in impairment of processes underlying metastasis development. It cannot be excluded that all the above-mentioned mechanisms could act together. It has been recently reported in an animal model, where mice with subcutaneous implantation of Lewis Lung Carcinoma were subjected to an operative injury, that surgery induced the release of cytokines/chemokines and mobilized bone marrow-derived cells [59]. These mobilized cells were then recruited into tumor tissue with concomitant enhancement of angiogenesis, thereby accelerating tumor growth. Furthermore, blocking recruitment of bone marrow stem cells by disrupting SDF/CXCR signals completely negated the accelerated tumor growth.

The mechanical and biological explanations may both be right. We suspect that for a few critical days post surgery, transient systemic inflammation triggers changes of the steady dormant status in the micrometastatic sites, where tumor cells together with their tumor associated cells lodge in metastatic niches or even may allow circulating tumor cells and cells released from surgery to enter tissue. These procedures result in relapses within the subsequent four years.

Are the missing early relapses in Forget et al. data never to happen or are they merely postponed to become late relapses? Whatever their source, cancer cells in circulation probably have half lives of a few days or less. Unless injected into more hospitable surroundings such as tissue, these cells will likely harmlessly die off. These data and our analysis suggest that at least for some patients the early relapses apparently avoided in the Forget et al. data do not show up later.

Perhaps ketorolac administered perioperatively is the right drug, at the right time, to prevent both surgery-driven angiogenesis and surgery-driven single cell activation. Indeed, the early relapses in the Brussels data are reduced by five-fold for patients treated with NSAID ketorolac compared to the other analgesics. We consider triple negative breast cancer, a poor prognosis group, to be an ideal subgroup with which to conduct a randomized clinical trial of ketorolac vs. a placebo control to test whether this observational study stands up to more robust scrutiny [36].

## Conclusions

Our findings suggest that most relapses occurring within 4 years may be induced by the effects of breast cancer surgery.

Possible mechanisms are surgery-induced angiogenesis of dormant avascular micrometastases and transient systemic inflammation which, via the upregulation and release of factors stimulating endothelial cells to proliferate and the activation of the so called “vascular niche” will stimulate tumor growth or even, in the presence of circulating cancer cells and cells released as a result of surgery, produce what has been called inflammatory oncotaxis.

We have found that peri-operative anti-inflammatory agents appear to abrogate the early hazard of recurrence and we estimate that such intervention could reduce breast cancer mortality by 25% to 50%.

High priority should be given to test this hypothesis in a randomized trial as it is implementable regardless of state of socio-economic development because expensive drugs, modern imaging facilities and advanced pathology services are not particularly relevant to implementing this simple change.

Also as noted by Wallace et al. [60], the racial disparity in breast cancer outcome is due primarily to deaths within the first few years after diagnosis providing an additional motivation to test at the earliest opportunity what we report here.

This needs to be confirmed and if found true would be an ideal inexpensive and non-toxic solution for a significant fraction of the early relapse problem in breast cancer. Now *that* sounds like translational research.

## Abbreviations

CEBH: Commission d'Ethique Biomédicale Hospitalo-Facultaire de l'Université catholique de Louvain; NSAID: Non-steroidal anti-inflammation drug; IL-6: Interleukin-6; CTC: Circulating tumor cells.

## Competing interests

Michael Retsky is on the Board of Directors of the Colon Cancer Alliance ( http://www.ccalliance.org) and has a patent pending for a method of treating early stage cancer.

Katharina Pachmann has a patent for the cell analysis method, termed MAINTRAC® analysis.

No other conflicts of interest are reported.

## Authors’ contributions

MR conducted the computer simulation, drafted the manuscript, and provided the “Mechanical Explanation”, RD provided Milan database, analyzed clinical data, and proposed the “Biological Explanation”, PF and MDe K conceived of the Brussels retrospective analysis, first presented Forget et al. data and provided these data, RR, MB, JSV, IG, WJMH and KP participated in the study. All authors read and approved the final manuscript.
